# Maternal engineered nanomaterial inhalation during gestation alters the fetal transcriptome

**DOI:** 10.1186/s12989-017-0239-8

**Published:** 2018-01-10

**Authors:** P. A. Stapleton, Q. A. Hathaway, C. E. Nichols, A. B. Abukabda, M. V. Pinti, D. L. Shepherd, C. R. McBride, J. Yi, V. C. Castranova, J. M. Hollander, T. R. Nurkiewicz

**Affiliations:** 1 Department of Pharmacology and Toxicology, Ernest Mario School of Pharmacy, Rutgers University, Piscataway, NJ USA; 2 Environmental and Occupational Health Sciences Institute, Piscataway, NJ USA; 3 Division of Exercise Physiology, West Virginia University School of Medicine, Morgantown, WV USA; 4 Mitochondria, Metabolism & Bioenergetics Working Group, West Virginia University School of Medicine, Morgantown, WV USA; 5 Immunity, Inflammation, and Disease Laboratory, National Institute of Environmental Health Sciences, Research Triangle Park, NC USA; 6 Toxicology Working Group, West Virginia University School of Medicine, Morgantown, WV USA; 7 Department of Pharmaceutical Sciences, West Virginia University School of Pharmacy, Morgantown, USA; 8 Department of Physiology, Pharmacology, and Neuroscience, Robert C. Byrd Health Sciences Center, West Virginia University School of Medicine, 1 Medical Center Drive, Morgantown, WV 26506-9229 USA

**Keywords:** Nanotechnology, Toxicology, Nanomaterial, Inhalation, Epigenetics, Maternal, Fetal

## Abstract

**Background:**

The integration of engineered nanomaterials (ENM) is well-established and widespread in clinical, commercial, and domestic applications. Cardiovascular dysfunctions have been reported in adult populations after exposure to a variety of ENM. As the diversity of these exposures continues to increase, the fetal ramifications of maternal exposures have yet to be determined. We, and others, have explored the consequences of ENM inhalation during gestation and identified many cardiovascular and metabolic outcomes in the F1 generation. The purpose of these studies was to identify genetic alterations in the F1 generation of Sprague-Dawley rats that result from maternal ENM inhalation during gestation. Pregnant dams were exposed to nano-titanium dioxide (nano-TiO_2_) aerosols (10 ± 0.5 mg/m^3^) for 7-8 days (calculated, cumulative lung deposition = 217 ± 1 μg) and on GD (gestational day) 20 fetal hearts were isolated. DNA was extracted and immunoprecipitated with modified chromatin marks histone 3 lysine 4 tri-methylation (H3K4me3) and histone 3 lysine 27 tri-methylation (H3K27me3). Following chromatin immunoprecipitation (ChIP), DNA fragments were sequenced. RNA from fetal hearts was purified and prepared for RNA sequencing and transcriptomic analysis. Ingenuity Pathway Analysis (IPA) was then used to identify pathways most modified by gestational ENM exposure.

**Results:**

The results of the sequencing experiments provide initial evidence that significant epigenetic and transcriptomic changes occur in the cardiac tissue of maternal nano-TiO_2_ exposed progeny. The most notable alterations in major biologic systems included immune adaptation and organismal growth. Changes in normal physiology were linked with other tissues, including liver and kidneys.

**Conclusions:**

These results are the first evidence that maternal ENM inhalation impacts the fetal epigenome.

**Electronic supplementary material:**

The online version of this article (10.1186/s12989-017-0239-8) contains supplementary material, which is available to authorized users.

## Background

The Barker Hypothesis [1], Developmental Origins of Health and Disease (DOHaD) [2], and fetal programming [3], all explore the relationship between the health of the gestational environment and fetal development and how this predisposes to future disease or sensitivities. Maternal prenatal health challenges such as nutrient deficiency, undernourishment, gestational diabetes and hypertension have been linked to an elevated risk for postnatal cardiovascular diseases [4]. Recently, maternal environmental toxicant exposures have become of prominent interest in relation to the impact of exposure on the fetal milieu and subsequent progeny health [5]. We have reported that maternal ENM inhalation impairs the ability of uterine arterioles to properly dilate, and this impacts litter health in the form of pup weight, number and gender distribution; as well as impaired microvascular function [21]. While these studies have focused on the maternal development of a hostile gestational environment and subsequent reduction in fetal nutrients, fetal epigenetic modifications may also occur. Conceptually, this relationship is not novel, but applications of environmental toxicants to the maternal-fetal models are. For example, bisphenol A [6] and air pollution [7] have been shown to negatively impact fetal outcomes. However, the impact of maternal ENM on fetal health and/or epigenetic modification are poorly understood.

Despite the ubiquitous inclusion of engineered nanomaterials in widespread applications, and their projected proliferation in human endeavors, the consequences of maternal ENM inhalation on the developing fetus and their impacts on future health are at best, vague, yet they are increasingly becoming a health concern. The prevalence of ENM covers an immense spectrum: surface coatings and additives in common consumer products (electronics, food, cosmetics), additives in industrial processes (advanced building materials, synthetic fuels), and components of clinical applications (diagnostics, drug delivery, implantable devices). It is widely recognized that throughout the ENM life cycle, the greatest risk for human exposure and subsequent health consequences begins with ENM inhalation, and is typically followed by systemic injuries. We have reported that pulmonary and systemic microvascular inflammation [29, 32] follow ENM inhalation exposure. Consistent with this, other systemic morbidities known to follow pulmonary ENM exposures include: inflammation/apoptosis [8, 9], macrovascular and microvascular dysfunction [10], atherogenesis [11], and organ level ischemia [12]. The developing fetus is equally a systemic target of numerous anthropogenic toxicants [13].

The impact of gestational ENM exposures on maternal and fetal health have been increasingly studied in the past decade. Adverse impact of ENM exposures on maternal health [14] and pregnancy [15, 16] have been reported in animal models. Teratogenic and embryo-lethal effects associated with ENM exposure have been shown [17]. The outcomes from several studies also highlight post-natal behavioral deficits [18, 19], cardiovascular [20, 21], renal [15], immune [22], reproductive [23, 24], pulmonary, and metabolic [20, 25] abnormalities.

Epigenetics, or the transient control of genes through DNA methylation or histone modification, is a recent area of intense focus by governmental agencies recognizing mechanistic links between environmental toxicants and gene expression [26]. These adverse maternal and fetal outcomes strongly reflect the potential risk of ENM exposure during pregnancy that may be linked. However, given the inherent physiological dependencies and complexities of developing and maintaining a healthy pregnancy, linking the mechanisms of pulmonary exposure and gestational effects remains very challenging. Given the magnitude of and the complexity these transgenerational effects, the most effective approach may be to initiate studies from the fetal epigenome and/or transcriptome. This is largely because fetal epigenetic outcomes resulting from maternal ENM exposure consequences may be caused by the creation of a hostile gestational environment [27], and/or the direct impact of ENM interacting with the developing embryo [13]. Because either of these possibilities would compromise health, the purpose of these studies was to identify epigenetic changes in cardiac gene expression within the maternally exposed F1 generations. We hypothesized that because maternal ENM inhalation lead to uterine microvascular dysfunction [21], this contributes to a hostile gestational environment, and altered fetal gene expression results. To test this, pregnant dams were intermittently exposed to nano-TiO_2_ aerosols during gestational days 5-19, and their litters were studied on GD 20.

## Methods

### Animal model

Sprague Dawley rats were purchased from Hilltop Laboratories (250-275 g female; 300-325 g male). All experiments were approved by the West Virginia University Animal Care and Use Committee and experiments adhered to the National Institutes of Health (NIH) Guide for the Care and Use of Laboratory Animals (8th Ed.). Rats were provided food and water ad libitum and housed in an AAALAC approved animal facility at the West Virginia University Health Sciences Center. Before mating, rats were acclimated for a minimum of 72 h, as previously described [20]. Pregnancy was verified by identification of the vaginal plug, after which, rats were randomly placed into one of two nano-TiO_2_ exposure groups. These two exposure groups were virtually identical and were created to generate a discrete tissue bank for RNA sequencing, or ChIP sequencing.

### Engineered Nanomaterial

Nano-TiO_2_ P25 powder was purchased from Evonik (Aeroxide TiO2, Parsippany, NJ), containing anatase (80%) and rutile (20%) TiO_2_. Nano-TiO_2_ was prepared by drying, sieving, and storing, as previously described [28, 29]. Nano-TiO_2_ aerosols were created with our aerosol generator (US Patent No. 8,881,997) [30]. Particle characteristics have been determined including the primary particle size (21 nm), the specific surface area (48.08 m^2^/g) [29, 31], and the Zeta potential (−56.6 mV) [32].

### Nano-TiO_2_ inhalation exposures

The nano-particle aerosol generator (US Patent No. 8,881,997) and whole-body inhalation exposure system used for the current study have been described extensively in previous studies [29, 31]. This collective exposure system consists of a vibrating fluidized bed, a Venturi vacuum pump, cyclone separator, impactor and mixing device, an animal housing chamber, and real-time monitoring devices with feedback control. Nano-TiO_2_ was aerosolized via a high velocity air stream passing through the vibrating fluidized bed and into the Venturi vacuum pump. The generated aerosols then entered the cyclone separated, which is designed to remove agglomerates > 400 nm at an input flow rate of 60 l/min of clean dry air before entering the exposure chamber.

Size distribution, mean aerodynamic diameter, and relative mass concentration of the aerosols were monitored in real time (Electrical Low Pressure Impactor (ELPI), Dekati, Tempere, Finland) while the particle size distribution was also measured in real-time with a Scanning Mobility Particle Sizer device (SMPS; TSI Inc., St. Paul, MN). These measurements were verified throughout a given exposure by collecting nanoparticle samples on filters, and making hourly gravimetric measurements with a microbalance. This approach was also used to collect samples for transmission electron microscopy.

Inhalation exposures were initiated on GD 5.78 ± 0.11 and lasted for 7.79 ± 0.26 days of gestation. Exposure days were not consecutive to decrease animal stress. Once the steady state nano-TiO_2_ aerosol concentration was achieved, exposure duration was adjusted to produce a daily calculated lung deposition of 31 ± 1.1 μg per day, and the cumulative, calculated dose was therefore 217 ± 1.0 μg. Lung deposition was calculated based on previously described mouse methodology, and normalized to rat weight and to pregnant rat minute ventilation using the equation: D = F⋅V⋅C⋅T, where F is the deposition fraction (14%), V is the minute ventilation based on body weight, C equals the mass concentration (mg/m^3^), and T equals the exposure duration (minutes) [29, 33]. The target concentration was 10 mg/m^3^ and the duration was 4-6 h/exposure (depending on the steady state concentration, as this was used to calculate the lung burden). The last exposure was conducted 24 h prior to sacrifice and experimentation. Control animals were exposed to HEPA filtered air only.

### Chromatin Immunoprecipitation (ChIP) sequencing

#### Isolation

**Fig. 1:**
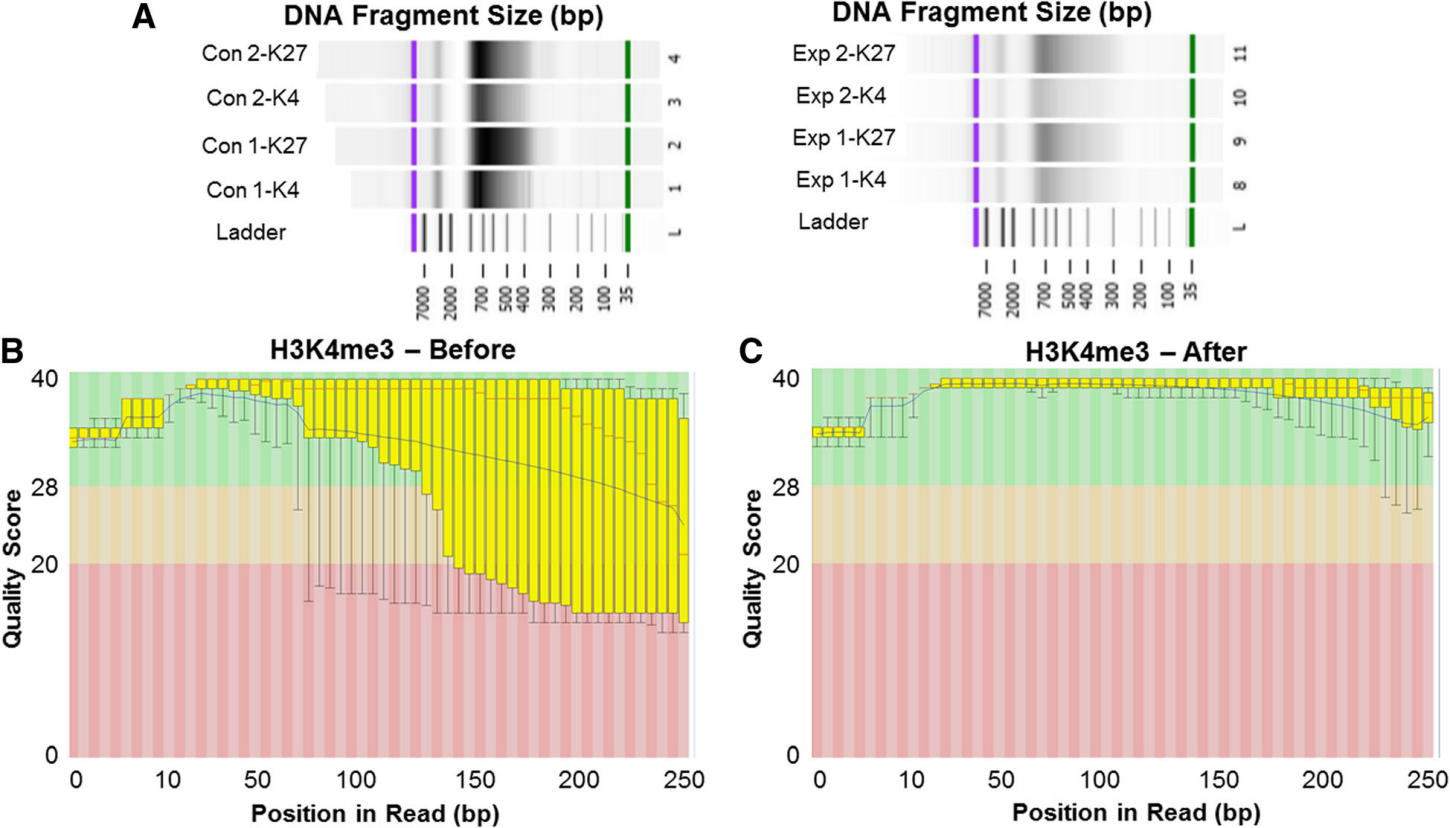
Evaluating DNA fragmentation and read quality for chromatin immunoprecipitation (ChIP) sequencing. **a** Using gel electrophoresis, DNA fragments were evaluated to determine size and distribution (average size of fragments = 654.3 bp). Two controls and two maternal nano-TiO_2_exposed representative samples are shown. Sample quality was assessed using FastQC for both forward and reverse reads (**b**) before and (**c**) after using Trimmomatic. Con = control, Exp = maternal nano-TiO_2_ exposed, H3K4me3 and K4 = histone 3 lysine 4 tri-methylation, K27 = histone 3 lysine 27 tri-methylation

Cardiac tissue was isolated from GD 20 pups in both the nano-TiO_2_ exposure and control groups. Each litter was considered an *n* = 1, with cardiac tissue from 5 to 6 pups within each litter being pooled together to collect enough tissue (~25 mg). Chromatin Immunoprecipitation (ChIP) was carried out using the MAGnify™ Chromatin Immunoprecipitation System (Thermo Fisher, Rockford, IL) per manufacturer’s instructions. Briefly, hearts were homogenized and treated with 37% formaldehyde, which was prepared fresh. Cross-linking was stopped with 1.25 M glycine. Samples were pelleted through centrifugation and washed in D-PBS before sonication. Using a Sonicator Ultrasonic Processor XL2015 (Misonix Sonicator, Farmingdale, NY) chromatin was sheared to a size of 500-700 base pairs, determined using gel electrophoresis (Fig. 1a). Chromatin was then isolated through ultracentrifugation (20,000 g) and diluted to ~60 uL of chromatin per immunoprecipitation reaction. Samples from both the control and nano-TiO_2_ cohorts were incubated with histone 3 lysine 4 tri-methylation (H3K4me3, product number: G.532.8, Thermo Fisher, Rockford, IL) or histone 3 lysine 27 tri-methylation (H3K27me3, product number: G.299.10, Thermo Fisher, Rockford, IL) antibody bound beads. These are two of the most prominently studied and classically applied for activation/repression analysis of gene activity. After incubation, samples were treated to reverse cross-linking solution and Proteinase K to remove bound proteins. DNA was then eluted from beads, using heat, and quantified using a Qubit (Thermo Fisher, Rockford, IL). The TruSeq ChIP Library Preparation Kit (Illumina, Inc., San Diego, CA) was implemented to build the libraries.

#### ChIP bioinformatics

Samples were processed using the Illumina MiSeq (Illumina, Inc., San Diego, CA) at the West Virginia University Genomics Core, ran as paired-end reads. Fastq files were assessed for quality using FastQC (Babraham Bioinformatics) (Fig. 1b), where it was determined that partial trimming was needed. Trimming of fastq files was accomplished through Trimmomatic [34] (Fig. 1c). Reads were then mapped to the rat genome (rn6) using the default parameters in bowtie2. To perform differential binding analysis on reads while distinguishing peaks, diffReps was used [35]. Bedtools functions were used to delineate upstream promoter regions of genes (bedtools slop) and evaluate the promoter/gene overlay (bedtools intersect). Genes were defined to include 1000 bases upstream from the start of the gene, indicative of our selected “promoter region.”

### RNA sequencing

#### Isolation

**Fig. 2:**
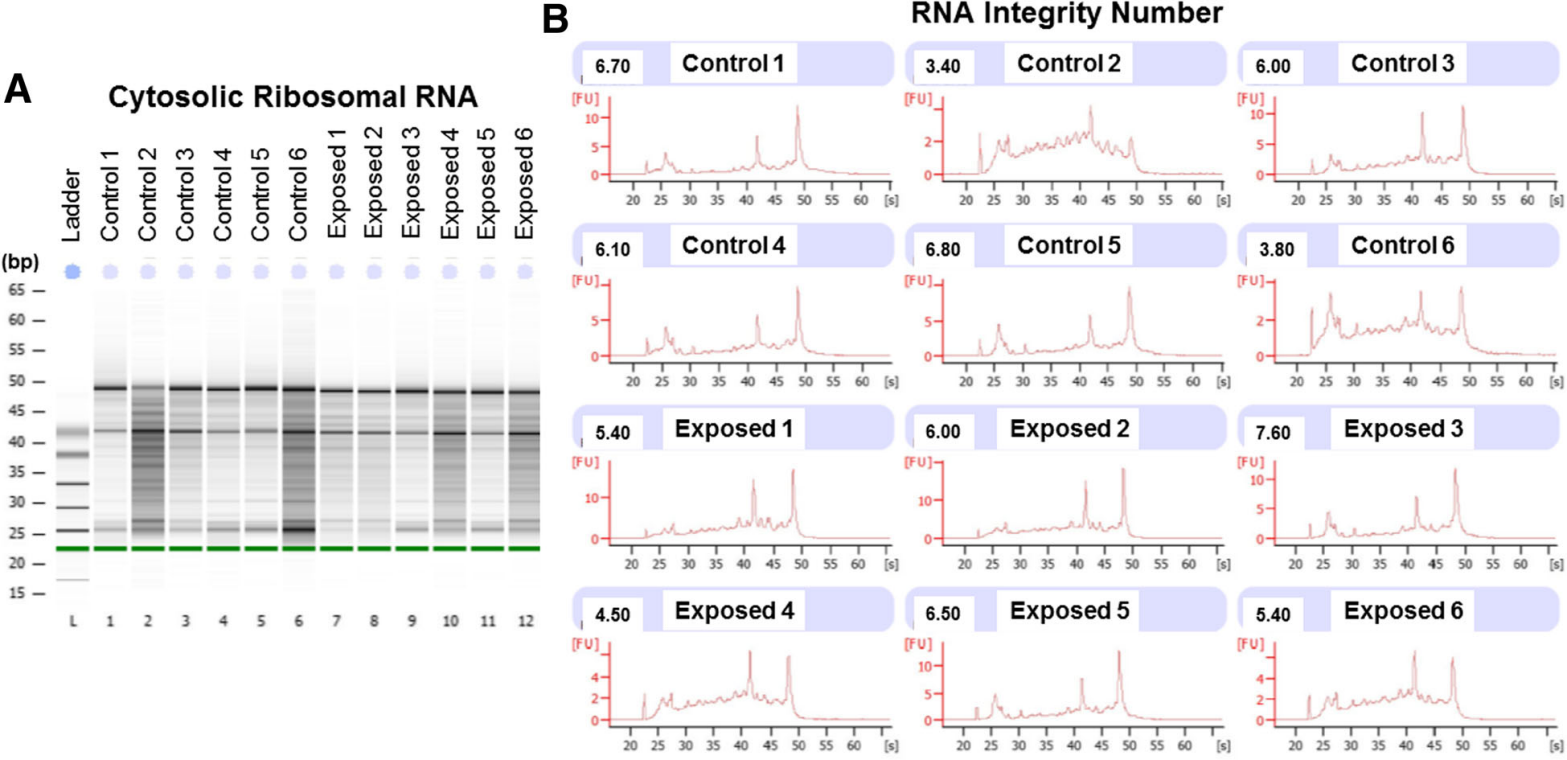
Assessing RNA quality for transcriptomic data. **a** Gel electrophoresis was implemented to visualize 28S and 18S ribosomal RNA quality. **b** Cytoplasmic, ribosomal RNA degradation was measured using the Agilent Bioanalyzer 2100. As determined by the RNA Integrity Number (RIN) (left of sample name) the five least degraded samples were chosen for the control (RIN = 5.88 ± 1.22) and exposed (RIN = 6.18 ± 0.92) groups. Exposed = maternal nano-TiO_2_ exposed

Cardiac tissue was procured through the same methods as listed above in the ChIP Sequencing section. RNA was then isolated from heart tissue using the Vantage™ Total RNA Purification Kit (Origene, Rockville, MD) per manufacturer’s instructions. Briefly, tissue was homogenized and lysis buffer was added to the sample. Sample RNA was spin-column purified and measured for RNA concentration using the Qubit (Thermo Fisher, Rockford, IL). Library preparation was performed using TruSeq RNA Library Prep Kit v2 (Illumina, Inc., San Diego, CA). Quality of RNA was determined using the Agilent 2100 BioAnalyzer (Agilent Technologies, Santa Clara, CA); degradation of cytosolic ribosomal RNAs (28S and 18S) are used as a measure of the total RNA Integrity Number (RIN) (Fig. 2a, b).

#### RNA bioinformatics

Samples were processed using the Illumina HiSeq (illumina, Inc., San Diego, CA) at Marshall University. Samples were run as paired-end reads. Paired-end, fastq files were aligned with HISAT2 [36] to the rat genome (rn6) without trimming. Samtools 1.2 [37] was used for the conversion of SAM to BAM format. Counts data was prepared using Subread 1.5.2 [38], specifically featureCounts [39]. Differential expression analysis was accomplished using DESeq2 [40] in R.

### Ingenuity pathway analysis (IPA)

Protein ontology and pathway analysis were completed through QIAGEN’s IPA (www.qiagen.com/ingenuity) software. Core analyses and comparative analyses were run on individual and combined ChIP and RNA data sets, respectively. Z-scores are representative of fold change between groups.

RNA IPA Protein Ontology.

The intensity of the color, moving toward blue or red, indicates the degree to which a specific pathway is being decreased or increased, respectively. The change in color, reflective of the z-score, is a quantitative measure of confidence (defined as the cumulative *P*-value of molecules in a specific pathway). This measure of confidence, defined on a color scale, indicates the propensity of all the molecules within that pathway to move in a certain direction, toward either increasing or decreasing the likelihood of developing the listed pathology or condition.

### Quantitative PCR

As described above, RNA was isolated from fetal heart tissue. Using the First-strand cDNA Synthesis kit for miRNA (Origene, Rockville, MD, Catalog #: HP100042), per manufacturer’s instructions, RNA was converted to cDNA. The cDNA was used for differential quantification of mRNA transcripts Fibroblast Growth Factor Receptor 1 (Fgfr1), Interleukin-18 (Il-18), and Transforming Growth Factor Beta Receptor 2 (Tgfbr2). ChIP-qPCR was used to assess the Tgfbr2 promoter loci. As described above, chromatin was immunoprecipitated with H3K4me3. DNA was then probed at multiple locations along the Tgfbr2 promoter region in order to construct a histone peak profile. Primer design for both the mRNA and ChIP-qPCR are provided (Additional file 1: Table S4). MRNA was normalized to Beta-Actin (β-Actin), while immunoprecipitated DNA was normalized to its respective input control. Experiments were performed on the Applied Biosystems 7900HT Fast Real-Time PCR system (Applied Biosystems, Foster City, CA), using 2X SYBR Green Master Mix. Quantification was achieved using the 2-ΔΔCT method.

### Statistics

All measures of significance between the control and maternal nano-TiO_2_ exposure groups for the sequencing data are presented as adjusted *P*-values. Adjusted P-values are a composition of standard, unadjusted P-values and the stringency of the False Discovery Rate (FDR). Differential expression analysis through DESeq2 implements the Wald Test, using multiple testing against the null hypothesis that P-values are uniformly distributed across a data set, known as the Benjamini-Hochberg procedure. The FDR for this study was set at 0.05. Z-score significance is determined as greater than the absolute value of 2. The z-score is computed as $z=\frac{x}{\sigma_x}=\frac{\sum_i{x}_i}{\sqrt{n}}=\frac{N_{+}-{N}_{-}}{\sqrt{N}}$, where N_+_ = the number of molecules following a consistent trend, N_−_ = the number of molecules following an inconsistent trend, and N = the number of interactions within a given pathway. In this way, the z-score, using only values with a significant change (*P* ≤ 0.05) can infer direction of a specific pathway while accounting for relationship and data bias and properly weighting the statistical findings (https://www.qiagenbioinformatics.com/products/ingenuity-pathway-analysis/). A consistency score is the non-statistical assignment of confidence to a specific pathway. Where appropriate, a Student’s t-test was used with all data presented as ± the standard error mean (SEM). Significance is determined as *P* ≤ 0.05.

## Results

### Animal and Nano-TiO_2_ aerosol characteristics

Animal number, age, body weight, and exposure conditions are provided (Table 1). Separate, but similar, inhalation exposures were used for the ChIP and RNA sequencing experiments. No statistical differences were noted between nano-TiO_2_ exposure in Experimental Group 1 (ChiP Seq) and Experimental Group 2 (RNA Seq). No statistical differences were noted in either progeny weight or total number of pups between maternal nano-TiO_2_ exposed or control groups.

**Table 1:** Animal characteristics

Exposure Group	Technique	Number of animals (N)	Age (days)	Body Weight (grams)	Litter Size	Pup Weight (grams)	Aerosol Concentration (mg/m^3^)	Electrical Low-Pressure Impactor (nm)	Scanning Particle Mobility Sizer (nm)
Sham Control	RNA Sequencing	7	109 ± 7	402 ± 8.84	13 ± 2	4.06 ± 0.16	0	0	0
Nano-TiO_2_	RNA Sequencing	4	113 ± 2	422 ± 13.34	14 ± 1	3.99 ± 0.22	10.35 ± 0.13	136.80 ± 1.44	134.80 ± 1.24
Sham Control	ChIP Sequencing	5	104 ± 2	407 ± 8.09	12 ± 2	5.19 ± 1.02	0	0	0
Nano-TiO_2_	ChIP Sequencing	6	98 ± 1	376 ± 19.99	9 ± 5	4.88 ± 1.53	10.5 ± 0.05	143.75 ± 2.32	129.43 ± 3.21

**Fig. 3:**
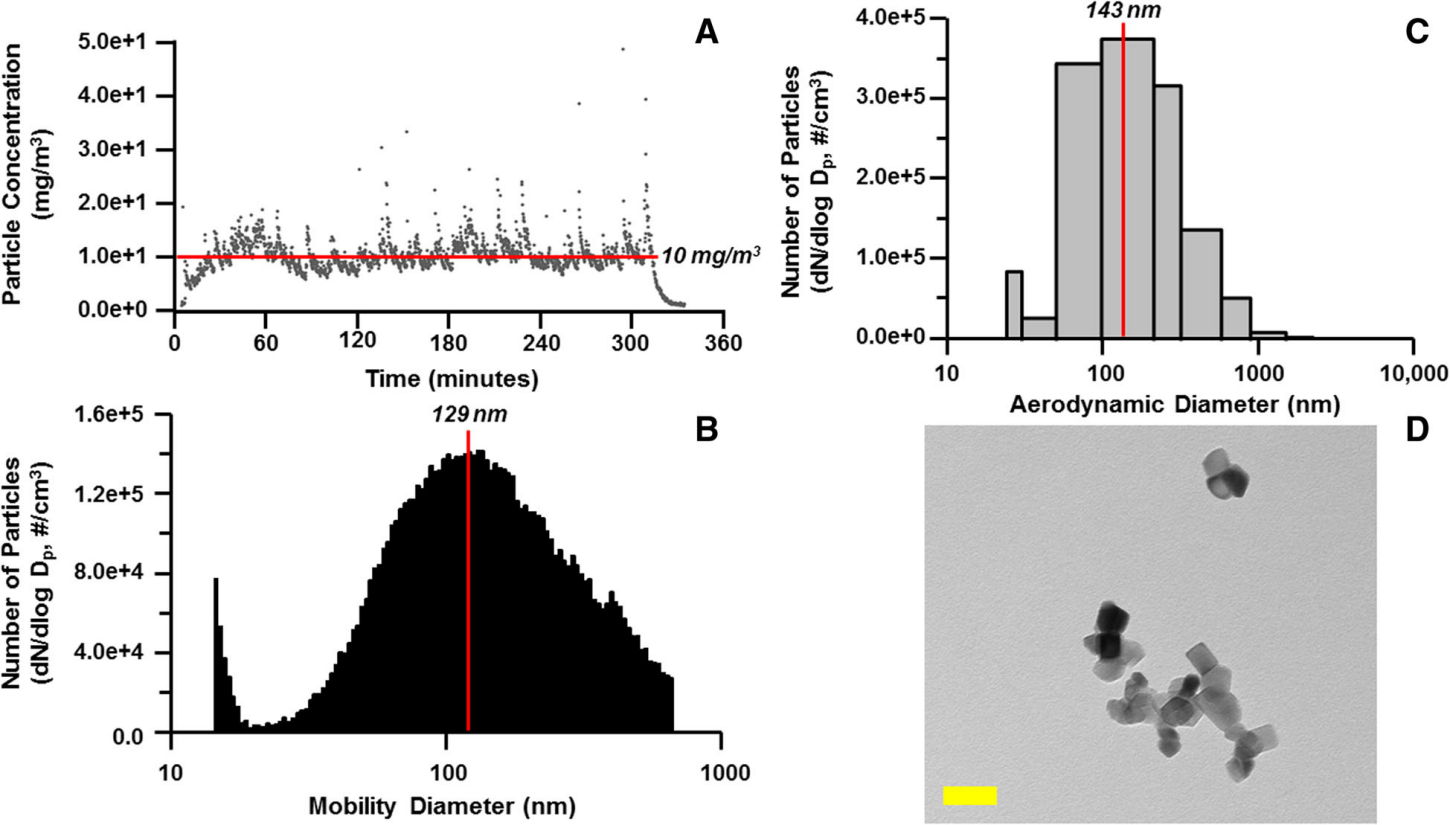
Maternal nano-TiO_2_ exposure particle characterization for RNA sequencing experiments. **a** Total aerosol concentration (10 mg/m^3^) of engineered nano-TiO_2_ during maternal exposures. **b** Nano-TiO_2_ size distribution (mobility diameter, 129.4 nm) using a scanning mobility particle sizer (SMPS). **c** Nano-TiO_2_ size distribution (aerodynamic diameter, 143.3 nm) using an electrical low-pressure impactor (ELPI). (D) Transmission electron microscopy image of aerosolized nano-TiO_2_ collected via a sampling filter during an exposure

Representative nano-TiO_2_ aerosol characterization data are presented in Fig. 3. The target particle concentration was 10 mg/m^3^ (Fig. 3a). The real-time nano-TiO_2_ mobility diameter was 129 nm (Fig. 3b), and the aerodynamic diameter was 143 nm (Fig. 3c). Nanoparticles were collected on filters, and a representative transmission electron microscopy image is presented in Fig. 3d.

### ChIP sequencing

#### ChIP sample metrics

**Fig. 4:**
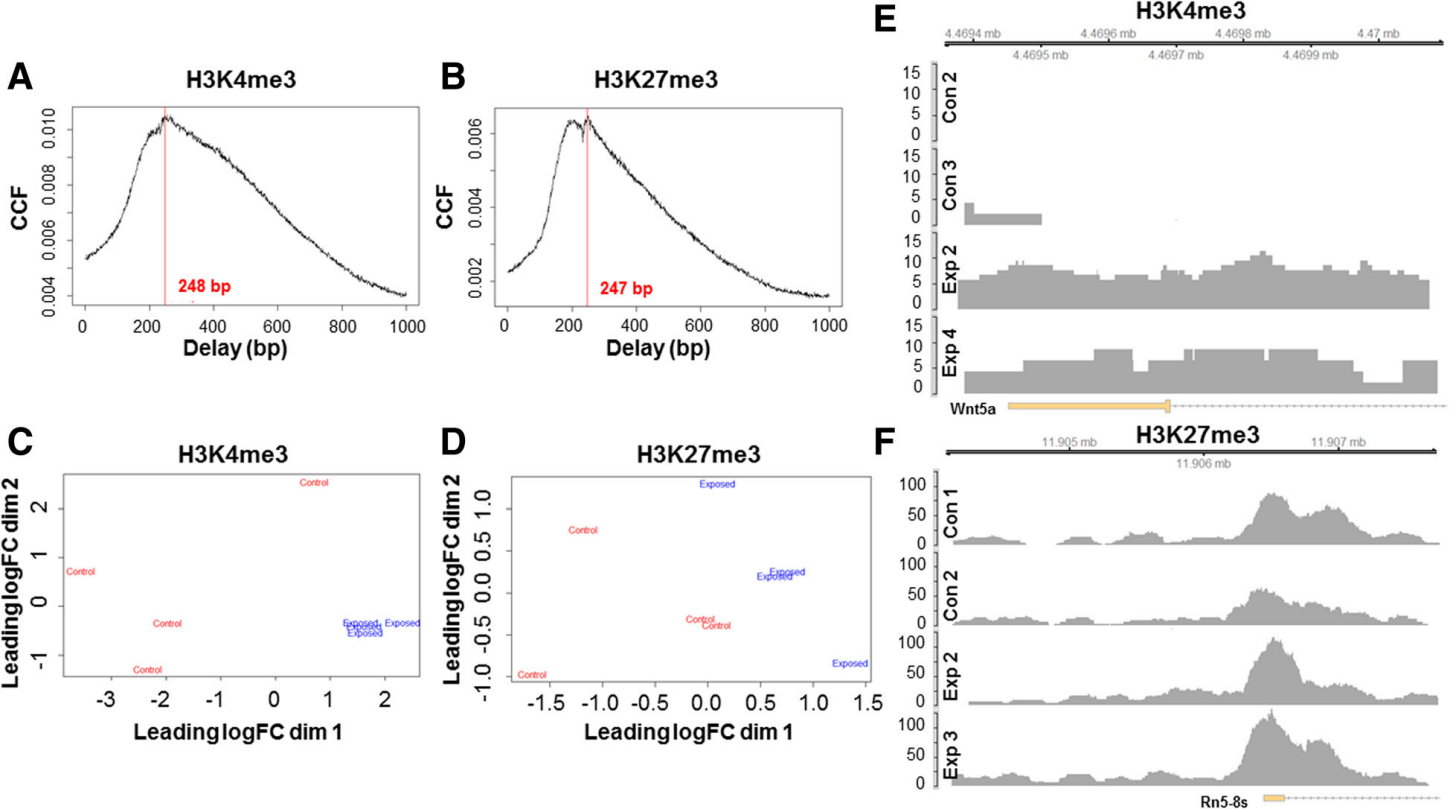
Chromatin immunoprecipitation (ChIP) sequencing fragment analysis and sample distribution. To measure the distance between subpeaks and find the maximum correlation, the cross-correlation function (CCF) was used to assess **a** H3K4me3 (248 bp) and (**b**) H3K27me3 (247 bp). Multi-dimensional scaling (MDS) plots indicate the log fold change (logFC) between samples within the (**c**) H3K4me3 and (**d**) H3K27me3 groups, describing sample-to-sample distances. Representative histone peaks are shown for differential binding regions (*P* ≤ 0.05) for both (**e**) H3K4me3 and (**f**) H3K27me3. Con = control, Exp = maternal nano-TiO2 exposed, H3K4me3 = histone 3 lysine 4 tri-methylation, H3K27me3 = histone 3 lysine 27 tri-methylation, Wnt5a = Wnt Family Member 5A, Rn5-8 s = 5.8S ribosomal RNA for *Rattus norvegicus*

To better understand the quality and sample dispersion within our cohort for the ChIP sequencing experiment, a series of statistical models were used. To assess the distribution of subpeaks present within the forward and reverse strands of the H3K4me3 and H3K27me3 immunoprecipitations, the average fragment length was determined for each event using the R package csaw [41]. The cross-correlation graph measures the delay distance, or number of base pairs, which separate distinctive subpeaks, also evaluating the consistency of fragment lengths within the data set (Fig. 4a and b). Multi-dimensional scaling (MDS) plots were used to evaluate individual library homology between both the H3K4me3 and H3K27me3 groups with the R package edgeR [42]. Log fold change (LogFC) determined the differences between libraries (control, red and maternal nano-TiO_2_ exposed, blue) within the MDS plots (Fig. 4c and d). To visualize read coverage, the R packages ChIPpeakAnno and Gviz were installed [43]. Complex, differential binding was assessed for both the H3K4me3 (Fig. 4e) and H3K27me3 (Fig. 4f) binding loci. Together, these results suggest that the immunoprecipitation and chromatin fragmentation were successful, and that differential binding is observed between groups.

#### ChIP IPA Protein Ontology

**Fig. 5:**
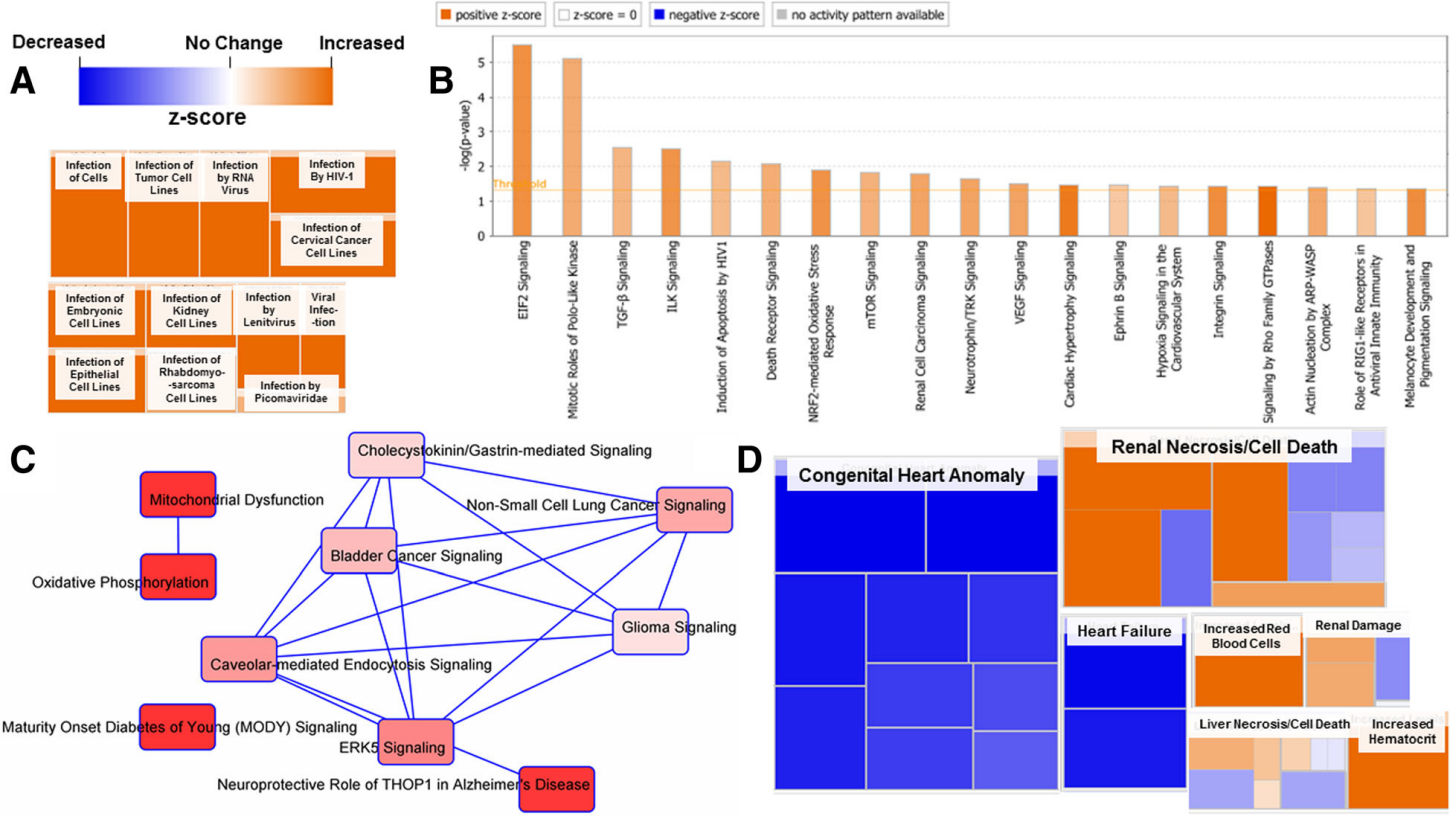
Assessment of disease and signaling pathways altered epigenetically during maternal nano-TiO_2_ exposure. **a** One of the primary disease pathways (z-score = 9.35 ± 1.89) altered epigenetically during exposure was the increased susceptibility to infection in the H3K4me3 group. Disease and toxicological pathways are constructed from specific, individual canonical signaling pathways. **b** Depicts the top canonical pathways for H3K4me3 (z-score ≥ ±2.0) that are significantly (*P* ≤ 0.05) impacted, as indicated by the threshold line. **c** The top canonical pathways for H3K27me3 (*P* ≤ 0.05) are also shown following exposure (smaller *p*-values are associated with increasing red intensity for pathways). **d** Toxicological functions predicted for genes mapped to H3K4me3 marks

Differential Binding data for both the H3K4me3 and H3K27me3 marks were uploaded and analyzed in QIAGEN’s IPA; all changes are shown as maternal nano-TiO_2_ exposed condition relative to the control. Diseases and biological functions (z-score ≥ 2) for H3K4me3 and H3K27me3 are provided in Additional file 1: Table S1 and S2, respectively. Of the diseases and biological functions listed, one of the most prominent pathways for H3K4me3 involved infectious disease (Fig. 5a). The heat map reveals how changes in molecular signaling could provide an increase susceptibility to infection in maternal nano-TiO_2_ exposed offspring. The top canonical pathways (z-score ≥ 2) altered during maternal nano-TiO_2_ exposure are presented (Fig. 5b). In general, the canonical pathways altered after exposure involve regulation of growth and cell cycle/apoptosis signaling.

For H3K27me3, the top 10 canonical pathways which are altered are provided (Fig. 5c). For the promoter regions associated with H3K27me3, the majority of signaling changes involve cancer and immunity. A heat map for the toxicological functions of the data representing H3K4me3 is also presented (Fig. 5d). The size and distribution of each major category is proportional to the z-score, which revealed three major organs affected: the heart, kidney and liver. Toxicological pathways associated with the heart, including congenital heart anomaly, heart failure, cardiac hypertrophy (not shown), and cardiac dysfunction (not shown), were found to be significantly decreased in the maternal nano-TiO_2_ exposed group. Conversely, toxicological pathways associated with the liver and kidney including, renal necrosis and cell death, liver necrosis and cell death, renal damage, and liver damage (not shown) were found to be increased. Also, an increase in red blood cells, and subsequently the hematocrit, were observed. Increases in H3K4me3 at promoter regions for infection capacity and growth signaling as well as loci involving kidney and liver dysfunction, suggests epigenetic regulation which could significantly alter an organism’s susceptibility to disease and potential pre-disposition to future insult. The lack of changes shown for H3K27me3 may suggest an alternative repressive mark implemented as the bivalent companion of H3K4me3.

### RNA sequencing

#### RNA sample metrics

**Fig. 6:**
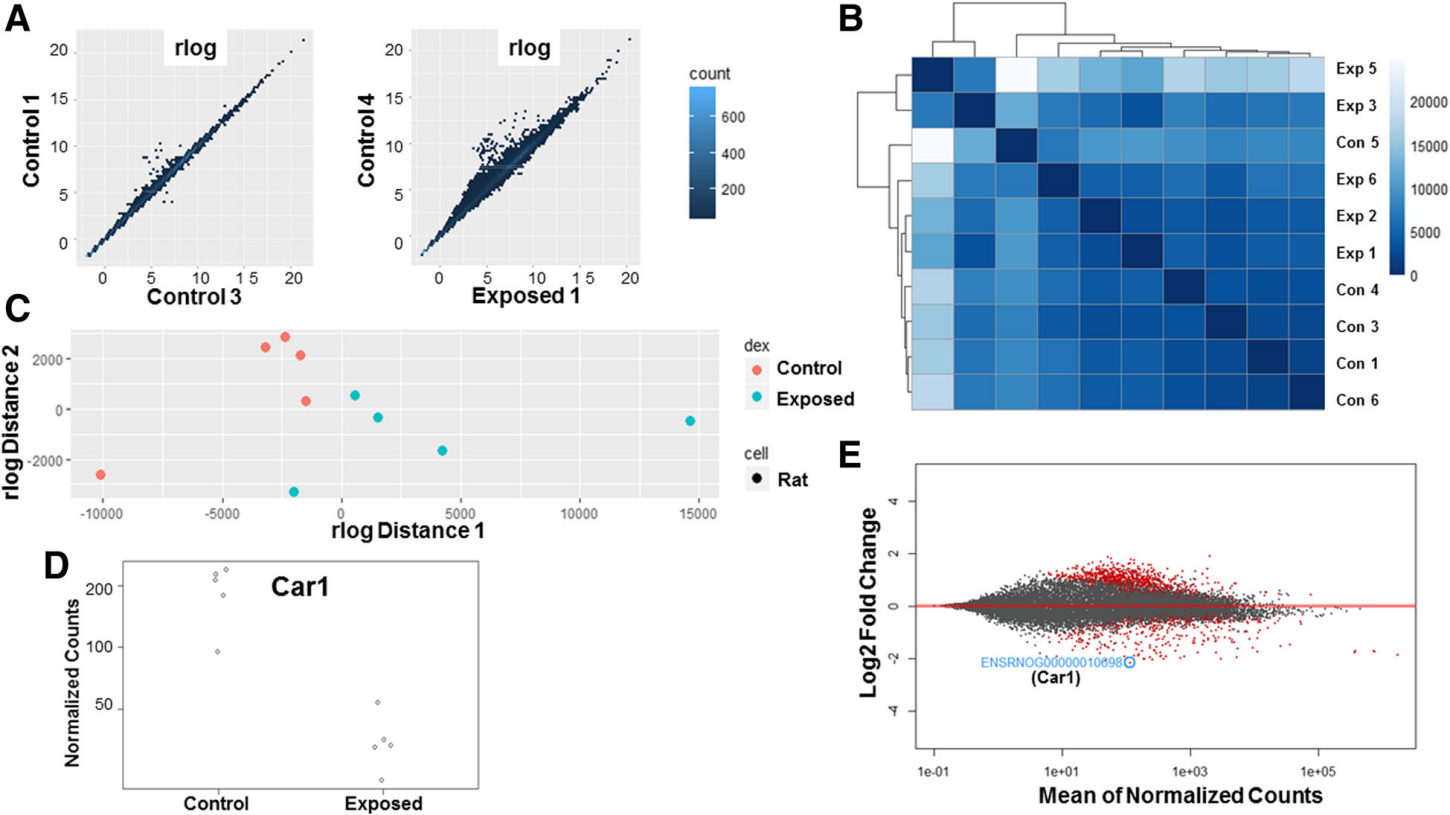
Sample-to-sample distribution and differential expression analysis for transcriptomic analysis. **a** Assessment of normalized counts between control vs. control (left) and control vs. maternal nano-TiO_2_ exposed (right) using a log2 transformed scale. **b** Measure of raw count matrices and (**c**) normalized count matrices to determine variance between samples. **d** The top differentially regulated gene between groups was determined through the normalized counts for each sample. **e** The MA-plot reveals the differentially expressed genes (red, *P* ≤ 0.05) in comparison to genes with non-significant change between groups (grey). The top differentially regulated gene is highlighted (blue). Exposed and Exp = maternal nano-TiO_2_ exposed, Car1 = carbonic anhydrase 1

The raw and normalized counts from the RNA sequencing experiment were subjected to a variety of statistical modelling, using the DESeq2 package in R [44], in order to better understand sample parameters. To visualize the variance of the normalized counts data means, the rlog function was used (Fig. 6a). For low-count genes, transformation using rlog, a log2 scale which normalizes data in reference to the library size, helps to better visualize variance-means. Fig. 6a shows limited outliers within the data set for the control vs. control, but increasing variance in the control vs. maternal nano-TiO_2_ exposed. Sample-to-sample distance was measured using the PoiClaClu package in R. Sample dissimilarity is depicted as a heat map (Fig. 6b), calculated from the original, not normalized count data. The heat map shows general dissimilarity between the maternal nano-TiO_2_ exposed and control groups, with the exception of one of the control samples. Another measure implemented for determining sample distance was a multi-dimensional scaling (MDS) plot based on the rlog-normalized counts (Fig. 6c). Again, the plot shows a general dissimilarity between the maternal nano-TiO_2_ exposed and control cohorts. After performing differential expression analysis with DESeq2, we examined the gene with the lowest associated *p*-value (Fig. 6d). The plot illustrates the similar expression of the gene within each group, while showing the disparities across groups. In Fig. 6e, a MA-plot is used to illustrate the number of genes (red) that fall below the *P*-value of 0.05. The statistical models used to assess the RNA sequencing samples indicate that normalized count values between groups are similar and that sample homology is close within groups, but not across groups.

#### RNA IPA protein ontology

**Fig. 7:**
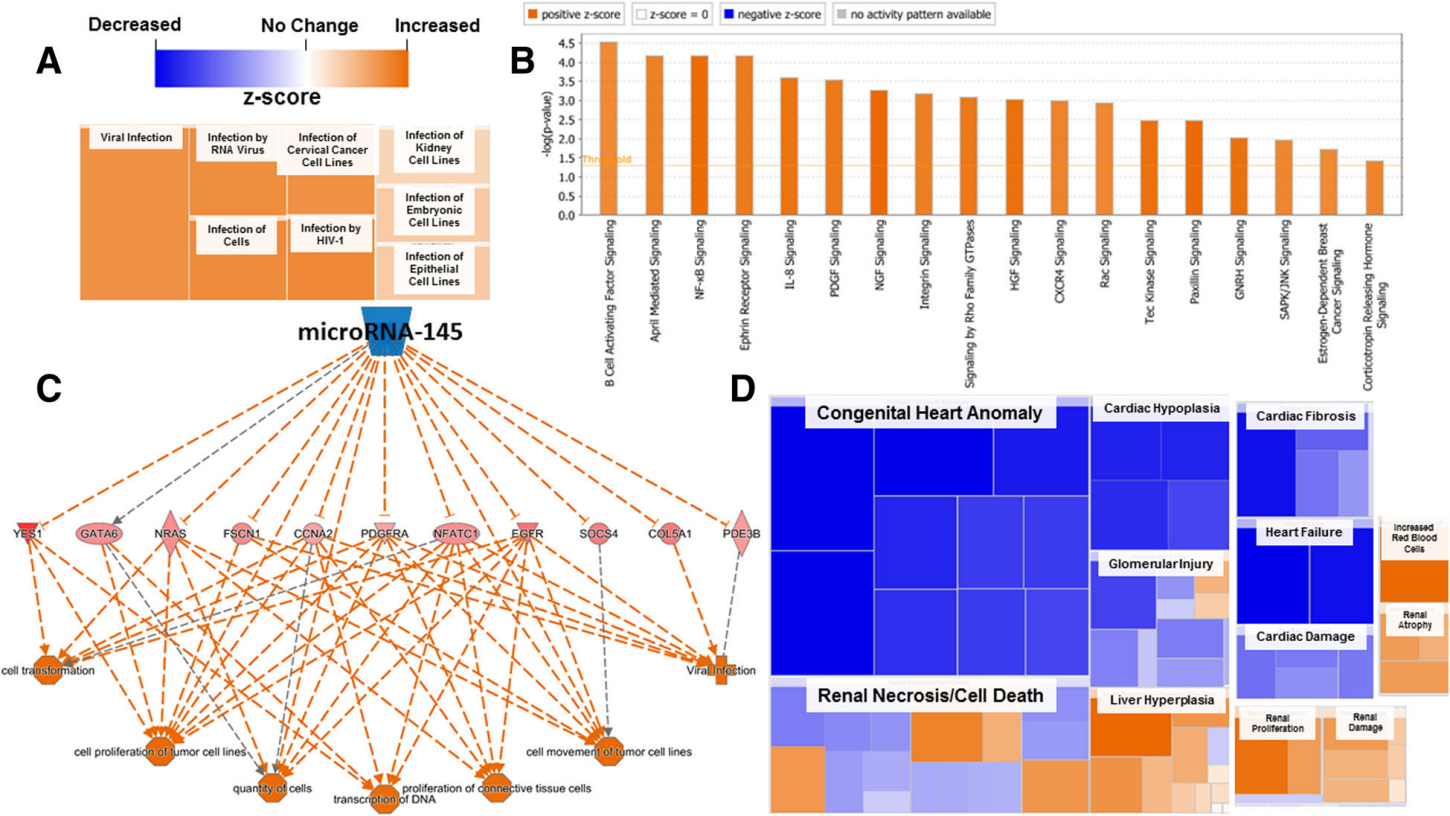
Assessment of disease and signaling pathways altered transcriptionally during maternal nano-TiO_2_ exposure. **a** Similar to the activation by H3K4me3, transcriptional upregulation of genes associated with increased susceptibility to infection (z-score = 2.02 ± 0.96) was found. **b** The top canonical pathways (z-score ≥ ±3.45) that are significantly (*P* ≤ 0.05) impacted transcriptionally, as indicated by the threshold line. The canonical pathways for the RNA sequencing reveal a significant increase in inflammatory and growth signaling. **c** The top regulator (consistency score = 10.453) determined through pathway analysis of gene expression (arrows = activation, bars = repression). Increasing gene activation (red) and suppression (blue) reveal targeting of multiple cell functions. **d** Toxicological functions predicted for transcript abundance in the RNA sequencing experiment

After differential expression analysis processing in R, data was uploaded and analyzed in QIAGEN’s IPA; all changes are shown as maternal nano-TiO_2_ exposed condition relative to the control. Diseases and biological functions (z-score ≥ 2) for the RNA are provided in Additional file 1: Table S3. Again, a prominent pathway that was found to be increased in the maternal nano-TiO_2_ exposed animals involved infectious diseases (Fig. 7a). Both the open promoter conformation (H3K4me3) and the RNA transcript expression reveal an increased propensity for infection. The top canonical pathways (z-score ≥ 3.45) altered during maternal nano-TiO_2_ exposure are presented (Fig. 7b). The canonical pathways altered primarily involve inflammatory signaling and organismal development. Examining what factors could be causing differential regulation after maternal nano-TiO_2_ exposure, we wanted to evaluate molecular regulator effects. The top molecule (consistency score ≥ 10.453) suggested to play a role in differential regulation of pathways was microRNA-145 (Fig. 7c).

In Fig. 7c, it reveals how decreased expression of microRNA-145 can lead to increased expression of pathways involving cell growth and proliferation. A heat map for the toxicological functions of the data representing the RNA is also shown (Fig. 7d). The size and distribution of each major category is proportional to the z-score and, again consistent with the H3K4me3 mark, three major organs were shown to be affected: the heart, kidney and liver. Toxicological pathways associated with the heart, including congenital heart anomaly, cardiac hypoplasia, heart failure, cardiac fibrosis, and cardiac damage, were found to be significantly decreased in the maternal nano-TiO_2_ exposed group. Alternatively, toxicological pathways associated with the liver and kidney including, renal necrosis and cell death, liver hyperplasia/hyperproliferation, renal proliferation, renal damage, and renal autophagy were found to be increased. As reported for the H3K4me3 promoter regions, increased RNA transcription of genes involving red blood production are shown. Similar to the epigenetic modification H3K4me3, the differential expression of transcripts follows a similar pattern of increased infection and growth of the organism, with increased molecular markers of dysfunction in the liver and kidney.

### Epigenetic regulation of transcription

**Fig. 8:**
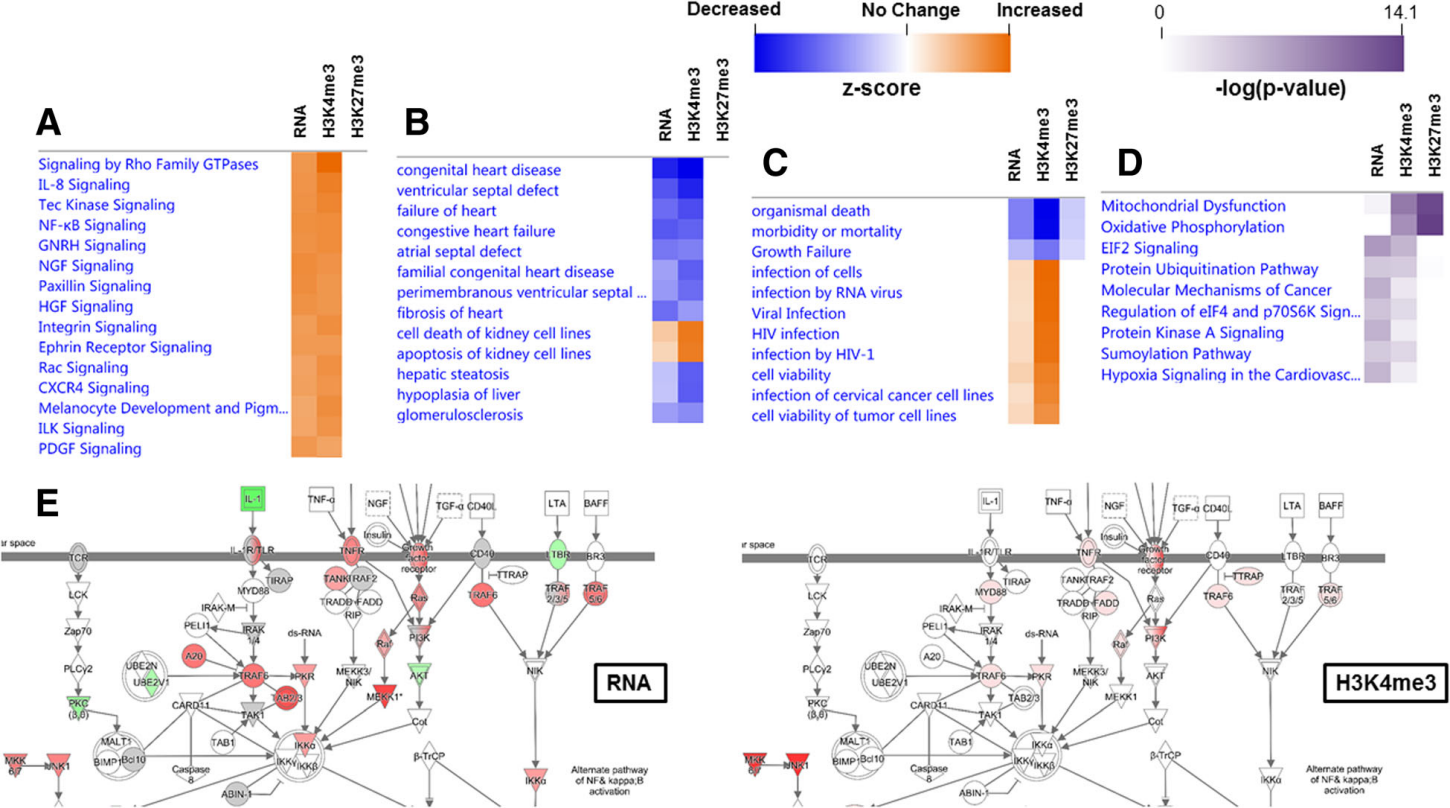
Comparison of epigenetic regulation (H3K4me3 and H3K27me3) and transcriptional changes. **a** Top canonical pathways, ranked by z-score, which are changed between groups. **b** Top toxicological functions, ranked by z-score, which are changed between groups. **c** Top diseases and biological functions, ranked by z-score, which are changed between groups. **d** Top canonical pathways, ranked by cumulative *P*-value, which are changed between groups. **e** Example of one of the top canonical pathways altered during maternal nano-TiO_2_ exposure. NF-ĸB signaling changes transcriptionally (right) and epigenetically through H3K4me3 (left) (green = decreased expression, red = increased expression). NF-ĸB = nuclear factor kappa-light-chain-enhancer of activated B cells

In order to examine how changes between the H3K4me3 mark and RNA transcript data aligned, we performed a comparative analysis through QIAGEN’s IPA, all changes are shown as maternal nano-TiO2 exposed condition relative to the control. The top canonical pathways (z-score ≥ 4.5) for both the transcript and ChIP data are shown (Fig. 8a). The combined data sets illustrate the common pathways involving both inflammation and organismal growth signaling. For toxicological functions, the molecular profile for cardiac dysfunction is significantly decreased compared to the controls, while kidney dysfunction is increased (Fig. 8b). A heat map for the cumulative diseases and biological functions is shown (Fig. 8c). The heat map depicts two major molecular changes that could impact the phenotype: increased survival and increased susceptibility to infection. In Fig. 8d, canonical pathways are sorted by *p*-value, depicting pathways with large sets of molecules having significantly altered expression levels. Although, the mitochondrial dysfunction and oxidative phosphorylation pathways do not have significant z-scores and a very small contribution of changes coming from the transcript data, Fig. 8b demonstrates the epigenetic changes occurring at these loci to a large segment of genes. Figure 8e illustrates the NF-ĸB (Nuclear Factor kappa-light-chain-enhancer of activated B cells) signaling pathway for the RNA (right) and H3K4me3 (left) sequencing experiments. The comparative analysis suggests that maternal nano-TiO_2_ exposure can cause significant changes to how the development of the progeny takes place, changing the epigenetic landscape, which can directly affect transcript abundance.

### Molecular validation of sequencing

**Fig. 9:**
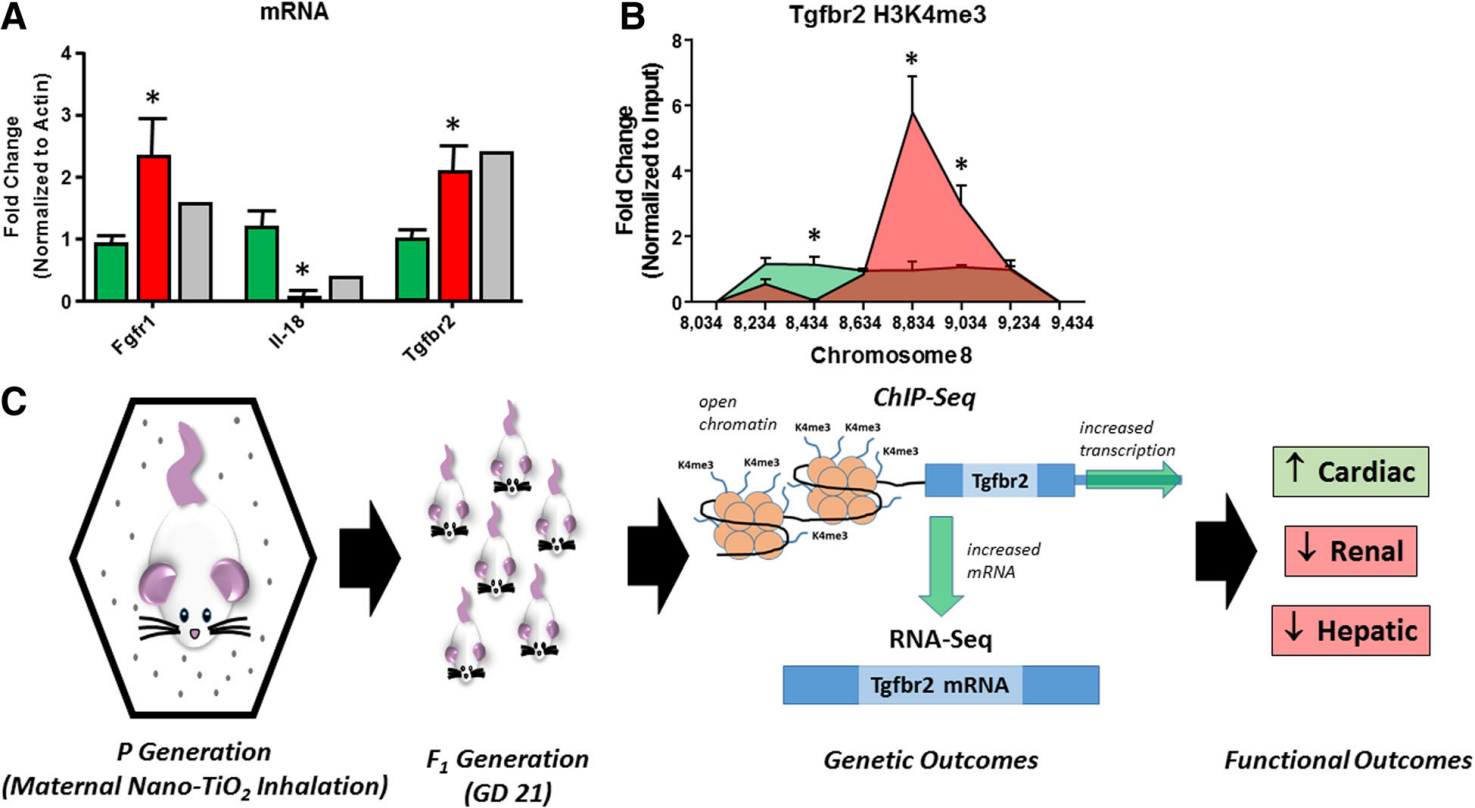
Validation of sequencing and model overview. **a** The mRNA of Fgfr1, Il-18, and Tgfbr2 were assessed in the sham (green, Sham-Control) and maternal nano-TiO_2_ (red, Nano-TiO_2_ Exposed) exposed progeny, reference to the RNA sequencing observed change (grey, Sequence). Expression was normalized to the β-Actin reporter gene. **b** Tgfbr2 was further characterized through ChIP-qPCR of H3K4me3 to measure the binding affinity of the modified histone at the Tgfbr2 promoter loci in the Sham-Control (green) and maternal nano-TiO_2_ (red) exposed progeny. Values were normalized to each sample’s input control. Tick marks represent the chromosomal location of each qPCR measurement, ranging from 124,318,034 to 124,319,434 on chromosome 8. **c** Schematic overview of the experimental model for nano-TiO_2_ maternal exposure and examination of the fetal progeny. As an example, the changes in Tgfbr2 are used to illustrate how epigenetic alterations through modification of chromatin can lead to increased expression of the mRNA transcript. Finally, the results of the study suggest that the gestational exposure paradigm impacts the heart, through increased function, while the liver and kidney have a detriment in function. Values are expressed as means ± SE. * = *P* ≤ 0.05. Fgfr1 = Fibroblast Growth Factor Receptor 1, Il-18 = Interleukin-18, Tgfbr2 = Transforming Growth Factor Beta Receptor 2, H3K4me3 = histone 3 lysine 4 tri-methylation, ChIP = Chromatin Immunoprecipitation

To further confirm the reliability of the sequencing data, we implemented qPCR to examine molecules involved in the NF-ĸB Pathway, which are not shown in the illustrative Fig. 8d, e. The mRNA levels of Fgfr1, Il-18, and Tgfbr2 are reported, and coincide with similar expression profiles seen in the sequencing data (Fig. 9a). In Fig. 9a, the data obtained from RNA sequencing (grey bars) are used as a reference to validate the expression profile of the maternal nano-TiO2 group when running qPCR. Likewise, we also wanted to use ChIP-qPCR to validate that histone modifications were also reliably reported, with the ChIP-Seq revealing epigenetic changes at the Tgfbr2 promoter region. We confirmed the H3K4me3 histone modifications for Tgfbr2, showing higher H3K4me3 association at its promoter region (Fig. 9b). The increased magnitude of the histone peak of the maternal nano-TiO2 group, Fig. 9b, suggests the increased abundance of H3K4me3 and active transcription of the Tgfbr2 gene. Tgfbr2 provides an explicit example of how genes reported to be epigenetically altered (ChIP-Seq, through H3K4me3 localization at the Tgfbr2 promoter region) with subsequent changes in transcription (RNA-Seq, reporting increased expression of Tgfbr2 transcripts) can be further validated using other molecular techniques, such as qPCR. An overview of the experimental design is illustrated in Fig. 9c. Briefly, the figure provides an example of suggested functional outcomes related to maternal nano-TiO2 exposure, with the link between the exposure paradigm and end function being fetal, epigenetic consequences.

## Discussion

The gene expression and epigenetic analyses performed in this study provide the first evidence that maternal ENM inhalation may result in significant pathway alterations in the fetus. The two most prominently impacted mechanisms are: inflammatory signaling, and cardiac-renal-hepatic pathology/toxicity.

The nano-TiO_2_ exposure paradigm used herein (10 mg/m^3^, 4-6 h) resulted in a calculated lung deposition of approximately 217 μg. This lung burden, achieved over 7 days of exposure in the second half of gestation, has been previously shown to impair uterine arteriolar reactivity by almost 50% [40]. To estimate how this lung burden compares to what a human may experience, alveolar surface areas must be known [32]. The rat alveolar surface area is 0.4 m^2^/lung. Therefore, the rat burden of 217 μg/lung would result in 542.5 μg/m^2^. Given that the human alveolar surface area is 102 m^2^, the equivalent human burden of this exposure paradigm would be 55.3 mg. The next logical question is how long would it take to achieve this burden in humans. In this regard, lung burden may be calculated as:

$$ nano-{TiO}_2\  aerosol concentration\cdot minute ventilation\cdot exposure duration\cdot deposition fraction, $$

with the following values:

$$ 55.3\  mg= nano-{TiO}_2\  aerosol concentration\cdot 7600\  ml/\mathit{\min}\cdot \left( 8\  hr/ day\cdot 60\ \mathit{\min}/ hr\right)\cdot 14\%, $$

and therefore:

$$ 55.3\  mg= nano-{TiO}_2\  aerosol concentration\cdot 0.51\ {m}^3/ day. $$

The National Institute for Occupational Safety and Health (NIOSH) Recommended Exposure Limit (REL), or aerosol concentration for nano-TiO_2_ is 0.3 mg/m^3^ (DHHS, 2011). This would result in a lung burden of 0.15 mg/day. Whereas, the Occupational Safety and Health Administration (OSHA) Permissible Exposure Limit is 5 mg/m^3^ (DHHS 2011). This would result in a lung burden of 2.55 mg/day. Considering the NIOSH REL and OSHA PEL together, it would require 1.45 working years or 21.7 working days (respectively) for a human to achieve comparable lung burdens with the exposure paradigm used herein. Because the human gestational period is 9 months, we consider our exposure paradigm highly relevant to the worker population.

Contrary to the functional deficits seen in the young adult [20, 25] we found that both the transcriptomic and epigenetic data support increased cardiac function (Figs. 5d and 7d). Though this seems paradoxical, we suggest that the interplay between the heart, liver, and kidneys is vital in understanding the pathology associated with maternal nano-TiO_2_ exposure. It is equally plausible that as hematocrit increases, viscosity of the blood also increases, requiring an elevation in contractile force or a drop in peripheral resistance. Alternatively, it is possible that disruptions in maternal-fetal perfusion balances occur. The pulmonary exposure of the mother is well described, but the secondary effect(s) on the developing progeny is/are likely to come through impacts on the maternal/fetal circulation. Maternal nutrients are delivered to the placenta via the arterial circuit, if blood flow is inadequate, then fetal compensation must occur to support proper nutrient delivery via the umbilical vein to the fetal portal circulation.

At the fetal stage, the heart plays a less significant role in energetics [45]. Whereas, the liver and kidneys play pivotal roles in blood conditioning at this stage of development, and these signaling pathways are altered by maternal ENM inhalation during gestation **(**Fig. 5**)**. We hypothesize that potential liver and kidney damage from either inflammation, direct ENM translocation or a combination may result in an increased hematocrit, and or maternal-fetal perfusion balance. Together, this may suggest that in maternal nano-TiO_2_ exposed progeny, the functional deficits seen later in development may be a result of this initial hepatic and renal insult, with subsequent cardiac overcompensation which may represent a protective mechanism. These findings correspond to reports of hepatic DNA damage in newborn murine offspring after maternal nano-TiO_2_ inhalation [46]. Impairments in renal function may have profound effects on tubuloglomerular feedback, the renin angiotensin system, and/or osmotic regulation. These impairments may collectively or individually directly influence cardiovascular health throughout prenatal and postnatal development.

MicroRNA (miRNA) are well known to be altered by transcriptomic and epigenetic regulators. When expressed, miRNA broadly regulate cellular function [47] and have been implicated in numerous epigenetic pathways [48]. In Fig. 7c transcriptomic data is provided that reflects the most consistently altered regulator after maternal nano-TiO_2_ inhalation. Decreased expression of miRNA-145 has been suggested to increase protein synthesis of targets directly involved in signaling events that promote organism growth and development. The role of altered miRNAs in progeny after maternal ENM inhalation is poorly understood, and may provide a better understanding of the relationship between ENM toxicities, epigenetics, and gene expression.

Figure 8c presents an overview of the two primary cell signaling pathways that are altered during gestational exposure: immunity and development. Parameters of organismal health and development are presented largely as molecular markers for cardiac signaling and function. The increased gene expression of molecular markers associated with infection and immunity may indicate the likelihood of autoimmune disorders associated with an overactive immune system. This is most evident when considering the inflammatory pathways indicated in Fig. 8a and the target organ (kidney) indicated in Fig. 8b reflected by an increased susceptibility as shown in Fig. 8c**.** These molecular markers may also represent the consequence presented in Fig. 8a of a proinflammatory environment; such an environment has been associated with chronic conditions including cardiovascular disease and cancer [49]. Pulmonary exposure to carbon black nanoparticles has also been identified to contribute to the development of immunotoxicity, particularly in lymphoid organs [22]. Interestingly, organismal death and morbidity/mortality appears to be decreased in maternal nano-TiO_2_ offspring, which may again seem counterintuitive. However, we speculate this may reflect a greater systemic response to compensate for the numerous other mechanisms disturbed by ENM inhalation during gestation.

To better identify the future consequences of ENM exposure, the significance of the pathways was represented as the change in *P*-value (Fig. 8d). Mitochondrial dysfunction and oxidative phosphorylation appeared to have the greatest changes in methylation, indicating that future complications in these pathways may occur. Given their widespread involvement, this epigenetic predisposition may manifest in any tissue. In other words, the epigenetic changes associated with energetics may reflect significant alterations that occur during fetal development. It is important to indicate that these changes may not be manifested in functional transcriptomic or proteomic changes until postnatal development or even later into adulthood. If correct, this would be consistent with the Barker Hypothesis and DOHaD.

Maternal nano-TiO_2_ exposure is also associated with a pronounced effect on key inflammatory pathways in the exposed progeny. In Fig. 8e, protein kinase B (AKT) signaling is decreased, potentially resulting in an impairment in calcium-independent nitric oxide signaling which would likely result in dysfunctional endothelium-dependent responses. Indeed, calcium dependent and independent mechanisms, as well as endothelial arteriolar dilation are significantly impaired at 3-4 weeks of age [50]. Furthermore, augmented NF-κB signaling via both alternate and canonical pathways [51] has been reported. Maternal nano-TiO_2_ exposure significantly activated the expression of the Lymphotoxin Beta Receptor (LTBR) gene, while suppressing the expression of the regulating enzyme Inhibitor of NF-κB Kinase Subunit Alpha (IKKα) Fig. 8e. This is important in the negative feedback of the NF-κB canonical signaling that limits inflammatory gene activation and suggests that more robust inflammatory responses are possible as evidenced in Fig. 8a**.** Furthermore, NF-κB plays a central role in the development of inflammation through further regulation of genes encoding not only pro-inflammatory cytokines, but also adhesion molecules such as E-selectin, VCAM-1 (vascular cell adhesion molecule-1) and ICAM-1 (intercellular adhesion molecule-1), chemokines, and inducible nitric oxide synthase (iNOS) [52, 53]. Figure 8e also reflects a significant increase in interleukin-8 (IL-8) signaling, a major chemokine associated with neutrophil chemotaxis and degranulation secreted by macrophages and endothelial cells during acute inflammatory responses [54]. Considered jointly, uncontrolled activation of NF-κB and IL-8 pathways in maternally exposed progeny may predispose towards endothelial-dependent dysfunction and leukocyte adhesion.

## Conclusion

The pathway analyses reported herein indicate dysfunction in many physiologic systems. As it is not possible to functionally verify each of these functional implications, the primary goal of the manuscript is to identify those systems as a priority for future study. Systemic impairments associated with acute and chronic nanomaterial exposures is an evolving field as nanotechnology continues to expand. Maternal and fetal outcomes following gestational exposures have recently been considered. While initial functional microvascular assessments have begun, little is known regarding epigenetic alterations within the F1 generation. The findings from this study describe epigenetic changes in the progeny of mothers exposed to nano-TiO_2_ aerosols during gestation. The evidence of the study is strengthened by the use of two separate cohorts to separately probe the transcriptomic and epigenetic alterations, suggesting that even in separate discrete experimental populations, changes to the epigenome and RNA transcript levels align and similar exposure paradigms yield consistent results. Changes in the RNA transcripts and histone modifications on DNA suggest that maternal nano-TiO_2_ progeny exhibit a propensity toward hepatic and renal disease, increased inflammatory signaling, and growth/survival while showing decreased cardiac dysfunction. What remains to be understood is if and/or how far these epigenetic changes persistent into adulthood, the dose-response relationships, and what stage of development is most sensitive to maternal ENM exposure.

## Additional file

Additional file 1: Table S1. Listing of all disease and biological pathways with a positive or negative z-score greater than the absolute value of 2 for H3K4me3. **Table S2.** Listing of all disease and biological pathways with a positive or negative z-score greater than the absolute value of 2 for H3K27me3. **Table S3.** Listing of all disease and biological pathways with a positive or negative z-score greater than the absolute value of 2 for RNA. **Table S4.** Primer design for qPCR experiments. Primers for the mRNA-qPCR experiments were designed using Primer-BLAST (https://www.ncbi.nlm.nih.gov/tools/primer-blast/). MRNA primers were designed to produced amplicons 100 – 250 bp in length. ChIP-qPCR primers were designed spanning a 1400 bp loci in the promoter region of the Tgfbr2 gene. Primers were designed to measure chromatin H3K4me3 modifications every 200 bp. Primer sequences were constrained to 60-100 bp amplicon lengths, in order to appropriately span sheered chromatin. All primer designs were performed against the *Rattus norvegicus* July 2014 (RGSC 6.0/rn6) genome build. ChIP = Chromatin Immunoprecipitation. (DOCX 41 kb)

## Abbreviations

AKT: Protein Kinase B
Car1: Carbonic anhydrase 1
ChIP: Chromatin Immunoprecipitation
DOHad: Developmental Origin of Health and Disease
ENM: Engineered Nanomaterials
FDR: False Discovery Rate
Fgfr1: Fibroblast Growth Factor Receptor 1
GD: Gestational Day
H3K27me3: 3 lysine 27 tri-methylation
H3K4me3: 3 lysine 4 tri-methylation
IACUC: Institutional Animal Care and Use Committee
ICAM-1: Intercellular Adhesion Molecule-1
IKKα: Inhibitor of NF-ĸB Kinase Subunit Alpha
Il-18: Interleukin-18
IL-8: Interleukin 8
IPA: Ingenuity Pathway Analysis
LogFC: Log Fold Change
LTBR: Lymphotoxin Beta Receptor
NF-ĸB: Nuclear Factor kappa-light-chain-enhancer of activated B cells
NOS: Nitric Oxide Synthase
PEL: Permissible Exposure Limit
qPCR: Quantitative Polymerase Chain Reaction
REL: Recommended Exposure Limit
RIN: RNA Integrity Number
Rn5-8 s: 5.8S ribosomal RNA for *Rattus norvegicus*
Tgfbr2: Transforming Growth Factor Beta Receptor 2
VCAM-1: Vascular Cell Adhesion Molecule-1
Wnt5a: Wnt Family Member 5A
