# Supplementary material for: Maternal engineered nanomaterial inhalation during gestation alters the fetal transcriptome
Source: Part Fibre Toxicol. 2018 Jan 10;15:3. doi: 10.1186/s12989-017-0239-8 (PMC5763571; doi:10.1186/s12989-017-0239-8)
Supplement: Additional file 1: Table S1. — Listing of all disease and biological pathways with a positive or negative z-score greater than the absolute value of 2 for H3K4me3. Table S2. Listing of all disease and biological pathways with a positive or negative z-score greater than the absolute value of 2 for H3K27me3. Table S3. Listing of all disease and biological pathways with a positive or negative z-score greater than the absolute value of 2 for RNA. Table S4. Primer design for qPCR experiments. Primers for the mRNA-qPCR experiments were designed using Primer-BLAST (https://www.ncbi.nlm.nih.gov/tools/primer-blast/). MRNA primers were designed to produced amplicons 100 – 250 bp in length. ChIP-qPCR primers were designed spanning a 1400 bp loci in the promoter region of the Tgfbr2 gene. Primers were designed to measure chromatin H3K4me3 modifications every 200 bp. Primer sequences were constrained to 60-100 bp amplicon lengths, in order to appropriately span sheered chromatin. All primer designs were performed against the Rattus norvegicus July 2014 (RGSC 6.0/rn6) genome build. ChIP = Chromatin Immunoprecipitation. (DOCX 41 kb) [file 12989_2017_239_MOESM1_ESM.docx]

**Supplemental Tables and Legends**

**Supplementary Table 1:** Listing of all disease and biological pathways with a positive or negative z-score greater than the absolute value of 2 for H3K4me3.

**Supplementary Table 2:** Listing of all disease and biological pathways with a positive or negative z-score greater than the absolute value of 2 for H3K27me3.

**Supplementary Table 3:** Listing of all disease and biological pathways with a positive or negative z-score greater than the absolute value of 2 for RNA.

**Supplemental Table 4:** Primer design for qPCR experiments. Primers for the mRNA-qPCR experiments were designed using Primer-BLAST (https://www.ncbi.nlm.nih.gov/tools/primer-blast/). MRNA primers were designed to produced amplicons 100 – 250 bp in length. ChIP-qPCR primers were designed spanning a 1,400 bp loci in the promoter region of the Tgfbr2 gene. Primers were designed to measure chromatin H3K4me3 modifications every 200 bp. Primer sequences were constrained to 60-100 bp amplicon lengths, in order to appropriately span sheered chromatin. All primer designs were performed against the *Rattus norvegicus* July 2014 (RGSC 6.0/rn6) genome build. ChIP = Chromatin Immunoprecipitation.

**Supplemental Table 1: H3K4me3 Disease and Biological Functions List**

| **Diseases or Functions Annotation** | **p-Value** | **Predicted Activation State** | **Activation z-score** |
| --- | --- | --- | --- |
| infection of cells | 0.000000955 | Increased | 11.372 |
| infection by RNA virus | 0.00000358 | Increased | 11.184 |
| Viral Infection | 0.00334 | Increased | 11.109 |
| HIV infection | 0.000494 | Increased | 10.705 |
| infection by HIV-1 | 0.0000125 | Increased | 10.623 |
| infection by lentivirus | 0.000392 | Increased | 10.613 |
| cell viability | 0.0094 | Increased | 9.722 |
| infection of tumor cell lines | 0.00000277 | Increased | 9.561 |
| infection of cervical cancer cell lines | 0.0000142 | Increased | 9.312 |
| cell viability of tumor cell lines | 0.0000388 | Increased | 8.467 |
| infection of kidney cell lines | 0.000339 | Increased | 7.151 |
| infection of embryonic cell lines | 0.00018 | Increased | 7.151 |
| infection of epithelial cell lines | 0.00018 | Increased | 7.151 |
| productive infection of cervical cancer cell lines | 0.00461 | Increased | 6.321 |
| cell proliferation of breast cancer cell lines | 0.0000852 | Increased | 5.014 |
| cell transformation | 0.00024 | Increased | 5.01 |
| cell proliferation of tumor cell lines | 0.00425 | Increased | 4.34 |
| homologous recombination of cells | 0.00141 | Increased | 4.252 |
| cell proliferation of fibroblasts | 0.00419 | Increased | 4.061 |
| cell viability of colorectal cancer cell lines | 0.00106 | Increased | 4.03 |
| cytokinesis | 0.00193 | Increased | 3.855 |
| repair of DNA | 8.61E-09 | Increased | 3.853 |
| metabolism of protein | 0.000352 | Increased | 3.587 |
| Clathrin mediated endocytosis | 0.00762 | Increased | 3.57 |
| development of cytoplasm | 0.00782 | Increased | 3.567 |
| transformation of fibroblast cell lines | 0.0133 | Increased | 3.548 |
| cell viability of myeloma cell lines | 0.00392 | Increased | 3.529 |
| recombination | 0.0101 | Increased | 3.475 |
| cell death of kidney cell lines | 0.000989 | Increased | 3.289 |
| formation of cytoskeleton | 0.0134 | Increased | 3.216 |
| apoptosis of kidney cell lines | 0.000769 | Increased | 3.21 |
| cell viability of breast cancer cell lines | 0.00257 | Increased | 3.181 |
| cytokinesis of tumor cell lines | 0.000305 | Increased | 3.148 |
| initiation of transcription | 0.0107 | Increased | 3.123 |
| excision repair | 0.00117 | Increased | 3.106 |
| proliferation of connective tissue cells | 0.00546 | Increased | 3.106 |
| interphase of fibroblasts | 0.00457 | Increased | 3.073 |
| interphase of connective tissue cells | 0.0121 | Increased | 3.066 |
| cell death of epithelial cell lines | 0.00656 | Increased | 2.992 |
| cell death of embryonic cell lines | 0.00184 | Increased | 2.99 |
| closure of embryonic tissue | 0.00665 | Increased | 2.97 |
| initiation of transcription of RNA | 0.0038 | Increased | 2.964 |
| apoptosis of embryonic cell lines | 0.00178 | Increased | 2.919 |
| apoptosis of epithelial cell lines | 0.011 | Increased | 2.87 |
| M phase | 0.000365 | Increased | 2.855 |
| growth of connective tissue | 0.013 | Increased | 2.852 |
| cytokinesis of cervical cancer cell lines | 0.000366 | Increased | 2.813 |
| expression of RNA | 0.00861 | Increased | 2.801 |
| transport of protein | 0.000134 | Increased | 2.743 |
| entry into interphase | 0.00904 | Increased | 2.704 |
| S phase | 0.00427 | Increased | 2.686 |
| cell death of kidney cells | 0.00148 | Increased | 2.681 |
| association of chromatin | 0.00108 | Increased | 2.606 |
| association of chromosome components | 0.0071 | Increased | 2.606 |
| modification of reactive oxygen species | 0.00717 | Increased | 2.502 |
| proliferation of fibroblast cell lines | 0.000267 | Increased | 2.465 |
| repair of cells | 0.00125 | Increased | 2.403 |
| necrosis of kidney | 0.00153 | Increased | 2.377 |
| initiation of expression of RNA | 0.00283 | Increased | 2.376 |
| apoptosis of nervous tissue cell lines | 0.00718 | Increased | 2.359 |
| M phase of cervical cancer cell lines | 0.00107 | Increased | 2.345 |
| cell cycle progression | 0.000099 | Increased | 2.268 |
| growth of yeast | 0.00836 | Increased | 2.266 |
| cell cycle progression of tumor cell lines | 0.0105 | Increased | 2.25 |
| checkpoint control | 0.000101 | Increased | 2.25 |
| infection of rhabdomyosarcoma cell lines | 0.000352 | Increased | 2.236 |
| modification of hydrogen peroxide | 0.00406 | Increased | 2.232 |
| catabolism of protein | 0.00263 | Increased | 2.231 |
| decay of RNA | 0.000808 | Increased | 2.213 |
| closure of neural tube | 0.00866 | Increased | 2.213 |
| maintenance of cells | 0.00741 | Increased | 2.209 |
| processing of rRNA | 0.0068 | Increased | 2.176 |
| ubiquitination | 0.0000226 | Increased | 2.15 |
| molecular cleavage of DNA | 0.00265 | Increased | 2.114 |
| condensation of chromatin | 0.0106 | Increased | 2.038 |
| cell viability of melanoma cell lines | 0.00287 | Increased | 2.034 |
| beta-oxidation of lipid | 0.014 | Increased | 2.028 |
| cell death of double-positive thymocyte | 0.00535 | Increased | 2 |
| hippocampal learning | 0.011 | Increased | 2 |
|  |  |  |  |
| **Diseases or Functions Annotation** | **p-Value** | **Predicted Activation State** | **Activation z-score** |
| organismal death | 0.000074 | Decreased | -20.188 |
| morbidity or mortality | 0.000068 | Decreased | -19.932 |
| Growth Failure | 0.00277 | Decreased | -11.033 |
| incidence of malignant tumor | 0.00892 | Decreased | -6.336 |
| cell death | 0.0000327 | Decreased | -6.293 |
| cell death of osteosarcoma cells | 0.000000464 | Decreased | -6 |
| Movement Disorders | 0.00954 | Decreased | -5.805 |
| midline defect | 0.00194 | Decreased | -5.446 |
| congenital anomaly of cardiovascular system | 0.00365 | Decreased | -5.027 |
| congenital malformation of brain | 0.00639 | Decreased | -4.978 |
| congenital anomaly of central nervous system | 0.00731 | Decreased | -4.978 |
| congenital heart disease | 0.012 | Decreased | -4.875 |
| incidence of lymphoma | 0.014 | Decreased | -4.829 |
| neural tube defect | 0.00998 | Decreased | -4.788 |
| kyphosis | 0.00517 | Decreased | -4.737 |
| apoptosis | 0.000114 | Decreased | -4.721 |
| exencephaly | 0.00568 | Decreased | -4.577 |
| ventricular septal defect | 0.000324 | Decreased | -4.231 |
| incidence of lung tumor | 0.0143 | Decreased | -4.042 |
| necrosis | 0.0069 | Decreased | -3.81 |
| non-melanoma solid tumor | 0.00000221 | Decreased | -3.115 |
| familial cardiovascular disease | 0.0142 | Decreased | -3.087 |
| damage of chromosomes | 0.00129 | Decreased | -2.874 |
| neoplasia of epithelial tissue | 0.00000394 | Decreased | -2.827 |
| perimembranous ventricular septal defect | 0.00492 | Decreased | -2.813 |
| cancer | 1.42E-08 | Decreased | -2.794 |
| Gastrointestinal Tract Cancer and Tumors | 0.0101 | Decreased | -2.745 |
| malignant solid tumor | 0.000000204 | Decreased | -2.697 |
| breakage of chromosomes | 0.00146 | Decreased | -2.65 |
| cell death of cancer cells | 0.0000006 | Decreased | -2.631 |
| necrosis of malignant tumor | 0.000000676 | Decreased | -2.631 |
| DNA damage response of cells | 0.0000451 | Decreased | -2.54 |
| tumorigenesis of tissue | 0.00000215 | Decreased | -2.507 |
| quantity of hydrogen peroxide | 0.00615 | Decreased | -2.483 |
| digestive organ tumor | 0.0000111 | Decreased | -2.482 |
| adenocarcinoma | 0.000662 | Decreased | -2.448 |
| liver carcinoma | 0.00825 | Decreased | -2.34 |
| hepatocellular carcinoma | 0.00872 | Decreased | -2.34 |
| epithelial cancer | 0.0000026 | Decreased | -2.255 |
| entry into interphase of cervical cancer cell lines | 0.00502 | Decreased | -2.236 |
| damage of protein | 0.00000992 | Decreased | -2.219 |
| abdominal neoplasm | 1.76E-08 | Decreased | -2.204 |
| cell death of tumor cells | 0.000000374 | Decreased | -2.195 |
| necrosis of tumor | 0.000000616 | Decreased | -2.195 |
| DNA damage response of tumor cell lines | 0.00247 | Decreased | -2.137 |
| digestive system cancer | 0.0000244 | Decreased | -2.084 |
| hepatobiliary system cancer | 0.0141 | Decreased | -2.056 |
| necrosis of brain cancer cell lines | 0.00298 | Decreased | -2.042 |

**Supplemental Table 2: H3K27me3 Disease and Biological Functions List**

| **Diseases or Functions Annotation** | **p-Value** | **Predicted Activation State** | **Activation z-score** |
| --- | --- | --- | --- |
| transcription of DNA | 8.38E-08 | Increased | 2.656 |
| activation of DNA endogenous promoter | 6.94E-09 | Increased | 2.572 |
| transcription of RNA | 0.0000023 | Increased | 2.483 |
|  |  |  |  |
| **Diseases or Functions Annotation** | **p-Value** | **Predicted Activation State** | **Activation z-score** |
| organismal death | 0.000264 | Decreased | -4.076 |
| morbidity or mortality | 0.000106 | Decreased | -3.87 |
| Growth Failure | 0.0000695 | Decreased | -3.132 |
| weight loss | 0.00158 | Decreased | -2.236 |

**Supplemental Table 3: RNA Transcript Disease and Biological Functions List**

| **Diseases or Functions Annotation** | **p-Value** | **Predicted Activation State** | **Activation z-score** |
| --- | --- | --- | --- |
| size of body | 0.000000139 | Increased | 7.093 |
| migration of tumor cell lines | 5.74E-08 | Increased | 4.915 |
| organization of cytoplasm | 6.32E-08 | Increased | 4.865 |
| organization of cytoskeleton | 0.0000006 | Increased | 4.865 |
| microtubule dynamics | 0.00000924 | Increased | 4.513 |
| cell movement of tumor cell lines | 7.56E-08 | Increased | 4.504 |
| cell death of osteosarcoma cells | 1.99E-09 | Increased | 4.32 |
| colony formation of cells | 0.0000099 | Increased | 3.903 |
| development of body trunk | 1.01E-10 | Increased | 3.896 |
| cell proliferation of tumor cell lines | 2.08E-10 | Increased | 3.77 |
| cell movement | 9.27E-15 | Increased | 3.71 |
| quantity of hematopoietic progenitor cells | 0.000000765 | Increased | 3.694 |
| quantity of cells | 5.04E-08 | Increased | 3.634 |
| migration of cells | 8E-14 | Increased | 3.563 |
| cell viability | 7.15E-12 | Increased | 3.526 |
| cell survival | 1.63E-12 | Increased | 3.513 |
| outgrowth of cells | 0.000041 | Increased | 3.435 |
| colony formation | 0.00000244 | Increased | 3.394 |
| cancer | 1.7E-41 | Increased | 3.346 |
| size of animal | 1.71E-08 | Increased | 3.342 |
| branching of cells | 0.0000051 | Increased | 3.193 |
| quantity of erythroid precursor cells | 0.0000343 | Increased | 3.146 |
| migration of endothelial cells | 2.65E-09 | Increased | 3.103 |
| cell movement of endothelial cells | 1.27E-09 | Increased | 3 |
| cell viability of tumor cell lines | 0.000000024 | Increased | 2.975 |
| quantity of heavy metal | 0.000000869 | Increased | 2.954 |
| synthesis of reactive oxygen species | 0.0000329 | Increased | 2.939 |
| transport of molecule | 0.0000013 | Increased | 2.898 |
| growth of neurites | 0.0000144 | Increased | 2.89 |
| cell proliferation of carcinoma cell lines | 0.0000533 | Increased | 2.873 |
| quantity of blood cells | 5.96E-10 | Increased | 2.837 |
| infection by HIV-1 | 0.0000122 | Increased | 2.82 |
| cell transformation | 2.87E-08 | Increased | 2.819 |
| HIV infection | 0.0000097 | Increased | 2.819 |
| hydrolysis of nucleotide | 0.0000349 | Increased | 2.778 |
| activation of DNA endogenous promoter | 1.13E-14 | Increased | 2.776 |
| development of head | 0.0000181 | Increased | 2.76 |
| movement of vascular endothelial cells | 0.00000163 | Increased | 2.749 |
| infection of cells | 0.00000573 | Increased | 2.74 |
| migration of vascular endothelial cells | 0.00000714 | Increased | 2.699 |
| transcription of RNA | 6.69E-19 | Increased | 2.678 |
| transport of metal | 0.0000371 | Increased | 2.662 |
| infection by RNA virus | 0.00000199 | Increased | 2.626 |
| invasion of cells | 0.000012 | Increased | 2.624 |
| sprouting | 3.93E-08 | Increased | 2.568 |
| transcription | 5.36E-17 | Increased | 2.559 |
| metabolism of reactive oxygen species | 0.00000685 | Increased | 2.524 |
| respiratory system development | 0.000000988 | Increased | 2.523 |
| formation of lung | 0.0000032 | Increased | 2.514 |
| infection of cervical cancer cell lines | 0.00000589 | Increased | 2.505 |
| transport of heavy metal | 9.84E-09 | Increased | 2.491 |
| development of body axis | 0.0000275 | Increased | 2.474 |
| differentiation of embryonic cells | 8.82E-08 | Increased | 2.468 |
| advanced malignant tumor | 0.0000156 | Increased | 2.459 |
| Viral Infection | 1.81E-13 | Increased | 2.455 |
| metastasis | 0.0000307 | Increased | 2.369 |
| necrosis of malignant tumor | 2.54E-10 | Increased | 2.307 |
| cell death of cancer cells | 7.85E-10 | Increased | 2.307 |
| quantity of connective tissue | 0.00000002 | Increased | 2.265 |
| quantity of red blood cells | 8.08E-08 | Increased | 2.198 |
| transcription of DNA | 7.81E-18 | Increased | 2.184 |
| development of vasculature | 1.79E-18 | Increased | 2.18 |
| thrombocytosis | 0.0000028 | Increased | 2.178 |
| angiogenesis | 1.06E-16 | Increased | 2.164 |
| spherocytosis | 3.06E-08 | Increased | 2.159 |
| proliferation of connective tissue cells | 4.28E-11 | Increased | 2.136 |
| necrosis of tumor | 9.88E-11 | Increased | 2.121 |
| cell death of tumor cells | 6.86E-10 | Increased | 2.121 |
| growth of connective tissue | 2.76E-10 | Increased | 2.118 |
| cell proliferation of fibroblasts | 4.96E-11 | Increased | 2.116 |
| expression of RNA | 1.9E-16 | Increased | 2.085 |
| transactivation | 2.22E-08 | Increased | 2.016 |
| transactivation of RNA | 5.26E-08 | Increased | 2.002 |
|  |  |  |  |
| **Diseases or Functions Annotation** | **p-Value** | **Predicted Activation State** | **Activation z-score** |
| organismal death | 5.59E-27 | Decreased | -10.051 |
| morbidity or mortality | 4.69E-27 | Decreased | -9.991 |
| perinatal death | 0.000000536 | Decreased | -6.598 |
| Bleeding | 1.41E-09 | Decreased | -5.689 |
| neonatal death | 0.00000164 | Decreased | -5.583 |
| Growth Failure | 0.00000029 | Decreased | -5.542 |
| Edema | 0.0000237 | Decreased | -5.48 |
| motor dysfunction or movement disorder | 0.000000629 | Decreased | -4.374 |
| Movement Disorders | 0.000000347 | Decreased | -3.935 |
| death of perinatal stage organism | 0.0000385 | Decreased | -3.328 |
| familial vascular disease | 0.0000265 | Decreased | -2.789 |
| hypoalbuminemia | 0.0000374 | Decreased | -2.449 |
| adhesion of cell-associated matrix | 0.0000211 | Decreased | -2.414 |
| senescence of cells | 0.000000606 | Decreased | -2.314 |
| adhesion of extracellular matrix | 0.0000059 | Decreased | -2.17 |
| congenital anomaly of central nervous system | 0.0000163 | Decreased | -2.144 |
| congenital encephalopathy | 0.0000293 | Decreased | -2.144 |
| congenital malformation of brain | 0.0000402 | Decreased | -2.144 |
| intestinal cancer | 3.74E-29 | Decreased | -2.066 |
| familial cardiovascular disease | 0.0000235 | Decreased | -2.035 |
| apoptosis | 7.86E-19 | Decreased | -2.012 |
| diffuse lymphoma | 0.00000118 | Decreased | -2 |

| **Supplemental Table 4. Primer Sequences** | | | |
| --- | --- | --- | --- |
| Experiment | Primer Name | Orientation | Sequence |
| mRNA-qPCR |  |  |  |
|  | Fgfr1 | F | GAAAGCAACCGTACACGCA |
|  | Fgfr1 | R | CGGTTTGGTTTGGTGTTGTCT |
|  | Il-18 | F | TCAGACCACTTTGGCAGACTT |
|  | Il-18 | R | CACAGGCGGGTTTCTTTTGTC |
|  | Tgfbr2 | F | ATCCTAGTAAGAAGCGATCTAACC |
|  | Tgfbr2 | R | GTCTGCTTGAAGGACTCCATGT |
| ChIP-qPCR |  |  |  |
|  | Tgfbr2 8043 |  | GTGTTTCACCCCAAATCCAC |
|  | Tgfbr2 8043 |  | ATCACTGGCTTTCCATGACC |
|  | Tgfbr2 8243 | F | GGACTGTCCTGCTCTTTTGC |
|  | Tgfbr2 8243 | R | CGGAAACGGGAAGTTTGTAG |
|  | Tgfbr2 8443 | F | AGCGAGGAGCCCTAGTGAAC |
|  | Tgfbr2 8443 | R | GTTCCCAAGTCGGGTGAGT |
|  | Tgfbr2 8643 | F | CCTTTATTCCTCGCCCTCTC |
|  | Tgfbr2 8643 | R | CTCCTGCGCAGCTCTCCT |
|  | Tgfbr2 8843 | F | GGGAGGGAAACAGGAAACTC |
|  | Tgfbr2 8843 | R | AGGAGAGGACCGGAGCTG |
|  | Tgfbr2 9043 | F | GGATTGGACCTTGAGGGACT |
|  | Tgfbr2 9043 | R | CGTGCGCTTAGTCACTTCTG |
|  | Tgfbr2 9243 | F | CAGGAGGGAGCTCTGAAATG |
|  | Tgfbr2 9243 | R | CGCATGCATGTCCATAAAAC |
|  | Tgfbr2 9443 | F | GGATTGGAAGGACCATTTCC |
|  | Tgfbr2 9443 | R | CGATCACAGGGCCAACTATT |
